# Critical thresholds and mediating mechanisms of ecological security in Shenyang based on health-risk-service triadic framework

**DOI:** 10.1016/j.isci.2026.116325

**Published:** 2026-06-14

**Authors:** Xiaofei Xu, Chunliang Xiu, Yanpeng Gao

**Affiliations:** 1 Northeastern University, School of Humanities and Law, 195 Chuangxin Road, Hunnan District, Shenyang, Liaoning, China; 2 Northeastern University, College of Architecture, 195 Chuangxin Road, Hunnan District, Shenyang, Liaoning, China

**Keywords:** Health sciences, Environmental science, Ecology, Risk assessment

## Abstract

Ecological security (ES) is a core pillar of China’s national security and underlies socioeconomic sustainability and human well-being. This study constructed a health-risk-service triadic framework, integrating machine learning with InVEST and Maxent models to explore critical thresholds and mediating mechanisms. Results showed that ecological resilience (ER) and landscape pattern (LP) were dominant driving factors with critical thresholds of 0.6 and 0.2, and their interaction presented a three-tiered continuum to reshape ecosystem service performance. Ecosystem health influenced ecosystem services primarily through direct effects, whereas ecological risk mediated these linkages through both partial mediating and masking effects. In particular, LP and natural disease (ND) exhibited relatively strong mediating roles. Practically, the suburban areas adopted two-phase management under the strategy of background safety maintenance and key window locking. The framework provides a quantifiable tool for sustainable land-use planning and ES management.

## Highlights

• Construct a health-risk-service framework combined with interpretable machine learning
• Clarify nonlinear threshold effects and interaction mechanisms of health and risk factors
• Explore mediating and masking effects of risk on the health-service correlation
• Put forward the LP-ER synergy window for spatial differentiated management in Shenyang

## Graphical abstract

**Figure:**
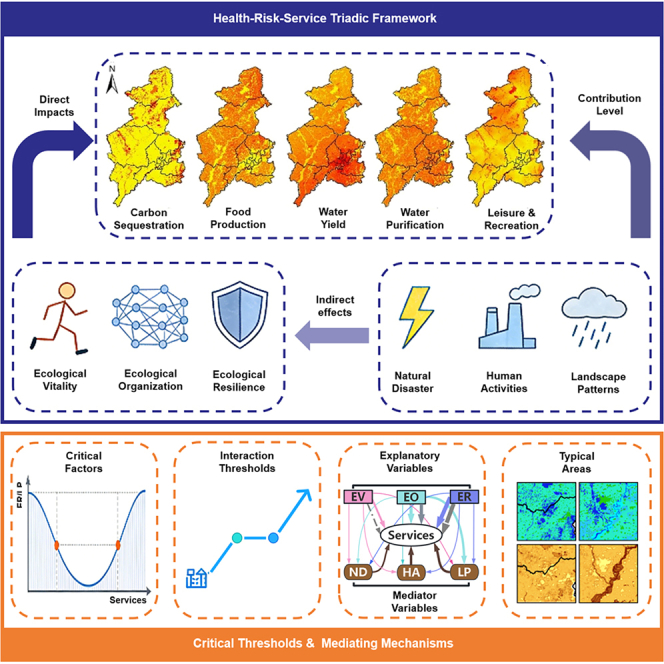

## Introduction

In the advancement of the national security system, ecological security (ES), as a vital component of the overall national security concept, has increasingly gained strategic prominence.^1^ Research on regional ES has also become an important means and a research hotspot for implementing macro-level ecosystem conservation and restoration, as it provides essential support for clarifying regional ecological patterns and formulating scientific protection strategies.

Generally, an ecosystem is considered secure when its structure and function are in a healthy state, it can stably provide products and services for human beings, and it operates within a low or acceptable risk range. This process involves three key interrelated characteristics: ecological risk, ecosystem health, and ecosystem services.^2^^,^^3^ Only when the management of these three aspects is mutually adaptive, coordinated, and interactive can regional ES be effectively guaranteed.^4^ Therefore, scientifically understanding the intrinsic relationships among ecological risk, ecosystem health, and ecosystem service functions, and revealing their underlying action mechanisms, are prerequisites and key links for effective ecosystem management. This is of great significance for safeguarding regional ES, promoting the sustainable development of society and economy, and enhancing human well-being.

Contemporary territorial ecological management relies on two key steps: integrated assessment of all territories and elements, and rigorous analysis of the mechanisms and multifaceted processes governing social-ecological systems (SESs). Against this methodological foundation, regional ecosystem management research is further tasked with two urgent priorities. One is to comprehensively clarify the complex mechanisms underlying the regulation of ecosystem services by multiple factors. The other is to achieve the efficient synergistic improvement of multiple ecosystem services.

To more effectively evaluate the integrated effects of human responses, social environments, and natural environments on ES, scholars worldwide have conducted extensive research on model construction, indicator systems, and evaluation methods. Examples include the PSR model^5^^,^^6^ and its derivatives, such as the DSR model,^7^ the DPSIR model,^8^^,^^9^ and the DPSIRM model,^10^ which are complemented by methods such as ecological footprint (EF), comprehensive indices, and the entropy matter-element model to assess ES. For instance, Chen established indicators to estimate regional ecosystem service value and ES by EF model.^11^ Kabubo-Mariara applied a comprehensive index method to assess land ES, analyzing the correlations among land tenure, land conservation, and population characteristics.^12^ Sun used the ecosystem service value equivalence factor method to calculate the ecosystem services.^13^ Despite their wide application, traditional models represented by the PSR framework have inherent limitations that hinder the in-depth exploration of ES mechanisms. First, these models are ineffective in capturing complex non-linear relationships between driving factors (e.g., land use change and climate fluctuation) and ES components. Second, they focus on static PSR indicator matching, failing to identify critical thresholds where ecological processes undergo abrupt changes—an essential basis for targeted management. Third, the interaction effects among multiple factors are either oversimplified or completely ignored in these models. For example, it remains unclear how ecological risk and health factors synergistically or antagonistically regulate ecosystem services. This oversight leads to a one-sided understanding of ES dynamics, rendering traditional assessments largely descriptive and static and unable to provide precise, threshold-based decision support for dynamic ecological management.

In this context, machine learning methods, with their powerful nonlinear modeling capabilities and ability to handle high-dimensional interactive data, have emerged as breakthrough tools to address the deficiencies of traditional models: They can automatically capture non-linear relationships without prior assumptions, quantify interactive effects among multiple factors through feature interaction analysis, and identify critical thresholds using partial dependence plots and break-point detection.^14^^,^^15^^,^^16^ Compared to traditional statistical methods, machine learning models offered superior predictive power and generalization performance, effectively capturing complex nonlinear relationships in data.^17^ However, the “black-box” nature of traditional machine learning models limited their application in management decision-making, as the lack of interpretability reduces decision-makers’ trust and willingness to adopt model results.^18^ The rise of interpretable machine learning methods addressed this issue by enhancing model transparency while maintaining predictive accuracy, clearly revealing key driving factors and their mechanisms.^19^ Techniques such as gray models, feature importance analysis, partial dependence plots, and SHAP values enabled in-depth analysis of the driving factors’ modes of action on ecosystem services. These methods demonstrated significant potential in ES prediction,^20^ ecosystem service trade-offs,^21^ and ecosystem health assessment.^22^ However, these approaches remain underutilized in comprehensively exploring the driving mechanisms of ES, especially in rapidly changing regional ecosystems, where methodological applicability requires further validation and expansion.

Besides, previous studies have widely confirmed that ecosystem health, as the foundation for maintaining structural integrity and functional stability, can effectively mitigate ecological risks caused by natural fluctuations and human disturbances.^23^^,^^24^ Meanwhile, the level of ecosystem health directly determines the supply capacity and sustainability of ecosystem services.^25^^,^^26^ Ecological risk can indirectly influence the provision and maintenance of ecosystem services by exerting the disturbance and constraints of ecosystem health.^27^^,^^28^^,^^29^ All of these indicate close hierarchical transmission and coupling linkages among the three components. However, existing research has mostly adopted the Health-Risk-Service framework to quantitatively investigate spatiotemporal evolution and external characteristics, while insufficient attention has been paid to indicator correlations and internal operational mechanisms, making it difficult to fully explain the internal logic of the ES system. The mechanisms through which ecosystem health influences ecosystem services remain largely unexplored. Equally unaddressed is how risk and health factors interrelate and jointly shape different types of ecosystem services.^30^^,^^31^^,^^32^ This paper aims to demonstrate that ecosystem services represent the ultimate outcome of both the direct impacts of ecosystem health and the indirect effects mediated by ecological risks in our integrated moderated mediation model, where ecosystem health impacts ecosystem services through a chain mediation of ecological risk.

**Figure 1:**
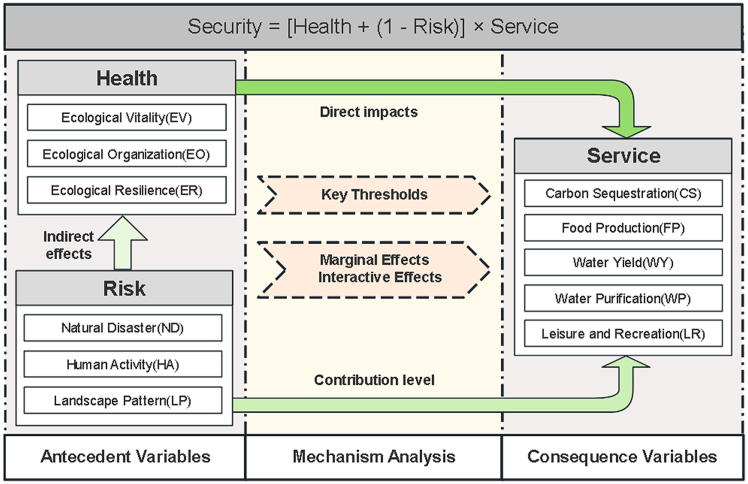
Mechanism conceptual framework

To fill the above research gaps, this study builds on the PSR model and integrates machine learning techniques (DT-RF) with the Health-Risk-Service triad framework to construct a dynamic ES assessment system. This integration realizes a paradigm shift from static indicator assessment to dynamic, threshold-oriented mechanism analysis and forms an integrated causal chain for regional ES. On this basis, the framework further leverages the advantages of machine learning in nonlinear fitting and interactive effect quantification to identify chain mediation. Specifically, it detects critical thresholds and interactive synergies that trigger abrupt changes in ecosystem services, clarifies how risk and health factors jointly regulate ecosystem services, and verifies the mediating role of ecological risk between ecosystem health and ecosystem services, thereby providing a more accurate decision-making basis for regional ecological management (Figure 1).

**Figure 2:**
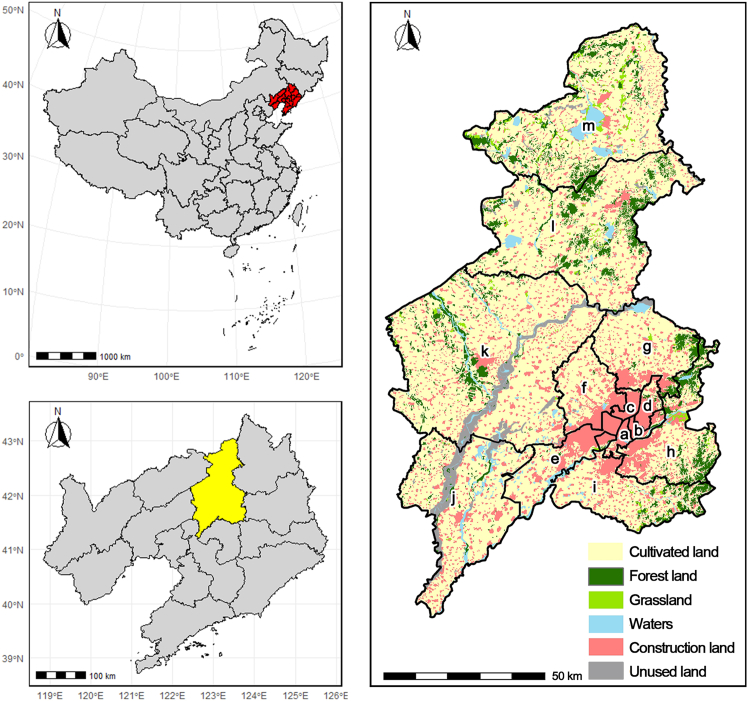
Study area and land use status in 2020 (a) Heping District, (b) Shenhe District, (c) Huanggu District, (d) Dadong District, (e) Tiexi District, (f) Yuhong District, (g) Shenbei New District, (h) Hunan District, (i) Sujiatun District, (j) Liaozhong District, (k) Xinmin City, (l) Faku County, and (m) Kangping County.

Therefore, this paper selects Shenyang City (Figure 2), a rapidly developing city in the Northeastern region that bears the dual responsibilities of social and economic development as well as food production (FP) and natural resource protection, as the research object. From the perspective of regional ES, this study quantitatively analyzes the spatial distribution characteristics of five types of ecosystem services in 2020, namely carbon sequestration (CS), food production (FP), water yield (WY), water purification (WP), and leisure and recreation (LR). More importantly, it employs interpretable machine learning methods to explore three key aspects: the thresholds, interactive effects, and impact pathways of health-risk factors on diverse ecosystem services. This approach directly addresses the core limitations of traditional models. By clarifying the internal relationships between health-risk-service in the security framework, this paper aims to enhance the understanding and awareness of regional ES and provide decision-making support for optimizing ecosystem management and safeguarding the ES of Shenyang City.

## Results

### Spatial distribution of ecosystem services and ecological security

**Figure 3:**
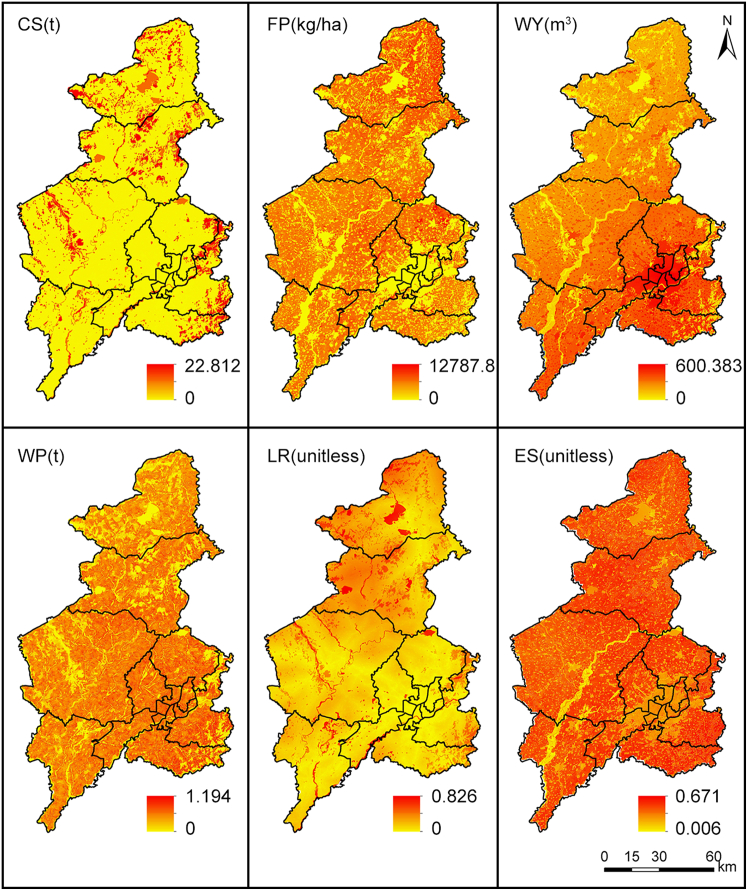
Spatial distribution of ecosystem service functions and ecological security in Shenyang

As shown in Figure 3, the five ecosystem service functions and ES in Shenyang exhibited significant spatial heterogeneity. CS aligned closely with land-use patterns, with high-value areas in eastern Shenyang and parts of Liaozhong, Xinmin, Faku, and Kangping counties, primarily overlapping with forest. Medium values were widespread in grassland and cultivated land, while construction land and waters had low CS value, averaging 4.6×10^6^ t. FP, exclusive to cultivated land, mirrored cropland distribution, with high-value areas concentrated in the counties and districts surrounding urban areas, with pronounced maxima located north of the city and in eastern Kangping County. The mean grain-production supply amounted to 7.6 × 10^3^ kg/ha.

The annual total WY capacity is 2.0 × 10^10^ m^3^, with high-value areas in central urban zones and low values in water bodies and forest zones, influenced by precipitation patterns. The spatial distribution pattern was influenced by precipitation distribution, exhibiting a characteristic of being low in towns and high in urban areas. Areas with superior WP capacity in Shenyang were concentrated in river basins and adjacent forest zones. Benefiting from abundant natural water sources and denser vegetation cover, these areas delivered markedly higher WP service. The average WP supply for the city in 2020 was 3.8 × 10^4^ t.

The distribution of LR in Shenyang was primarily influenced by natural conditions. There was a significant disparity in the supply capacity of scenic spot services, with high values primarily located in rivers and lakes such as the Liaohe River and Wolong Lake Reservoir, as well as natural protected areas and scenic spots such as Qipan Mountain Forest Park. These areas had a good ecological foundation, with a large distribution of ecological land and a wide radiation range. They also featured numerous river and lake surfaces, providing excellent venues and landscapes for people to enjoy scenic spots and recreation. The disparities in scenic spot service functions among different counties and districts were quite evident.

From the perspective of spatial distribution, the overall distribution characteristics of the ES in Shenyang were “high in the hilly areas in the northwest and southeast, low in the city center, and scattered low and medium values in the plains,” exhibiting significant regional heterogeneity. The heterogeneity was attributed to factors such as uneven regional development, spatial differences in land use types, and localized ecological restoration measures. High values of the ES were mainly distributed in the eastern part of the city and peripheral areas, such as ecological source regions in surrounding counties and districts, where the ecological environment quality was relatively superior, with the highest value reaching 0.671. Due to high-intensity land use and the reduction in ecological land in the central urban area, the ES was relatively low, with a minimum value of 0.006.

### Key thresholds of health-risk elements

Under the K-S test, the significance values for all Health-Risk factors were less than 0.001, indicating non-normality and a nonlinear relationship. The Spearman correlation coefficient was selected to measure the trade-offs and synergistic relationships in the ecosystem service functions and ES pattern of Shenyang. Additionally, this paper conducted a multicollinearity test on all the Health-Risk factors, finding that the variance inflation factor values ranged from 1.002 to 1.572, indicating that the selection of factors was reasonable and there was no multicollinearity issue (Table 1).

**Table 1:** Normality and collinearity test of health-risk factors in Shenyang city

Test parameters	Health			Risk		
	EV	EO	ER	ND	HA	LP
K-S Test						
significance	0.000	0.000	0.000	0.000	0.000	0.000
Collinearity test						
T value	1.587	11.733	1.194	17.087	−6.788	−3.245
*p* value	0.113	<0.001	0.233	<0.001	<0.001	0.001
VIF	1.177	1.014	1.469	1.042	1.046	1.388

**Figure 4:**
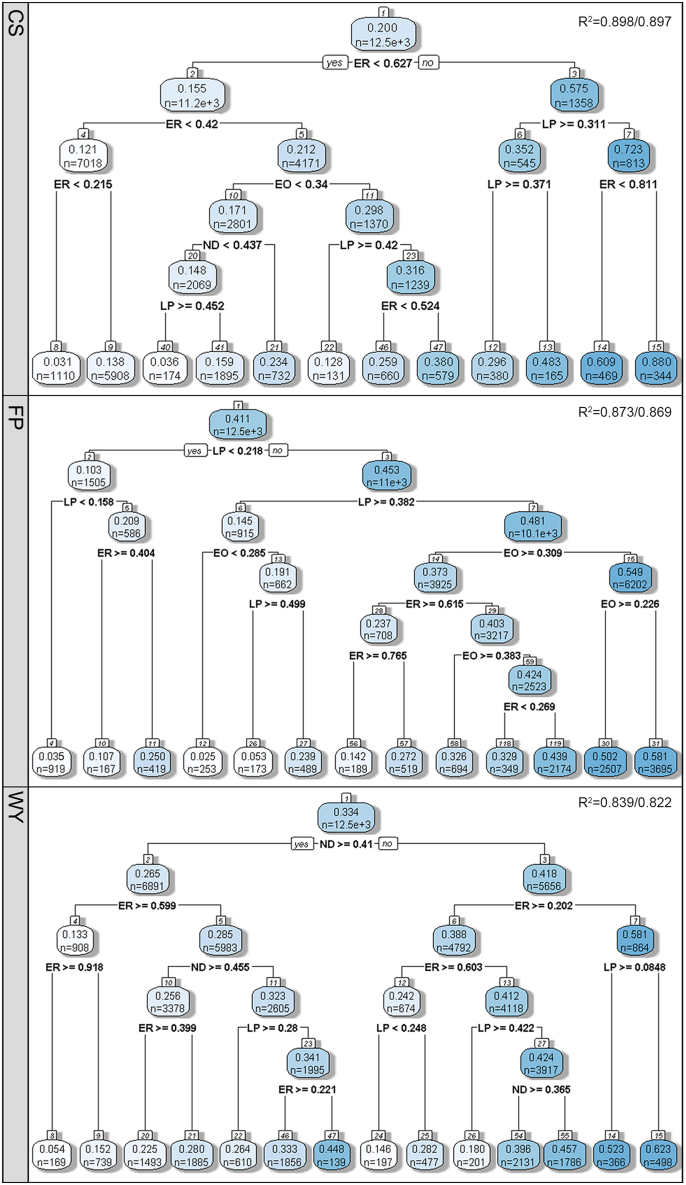
Decision trees for CS, FP, and WY with Health-Risk factors in Shenyang

**Figure 5:**
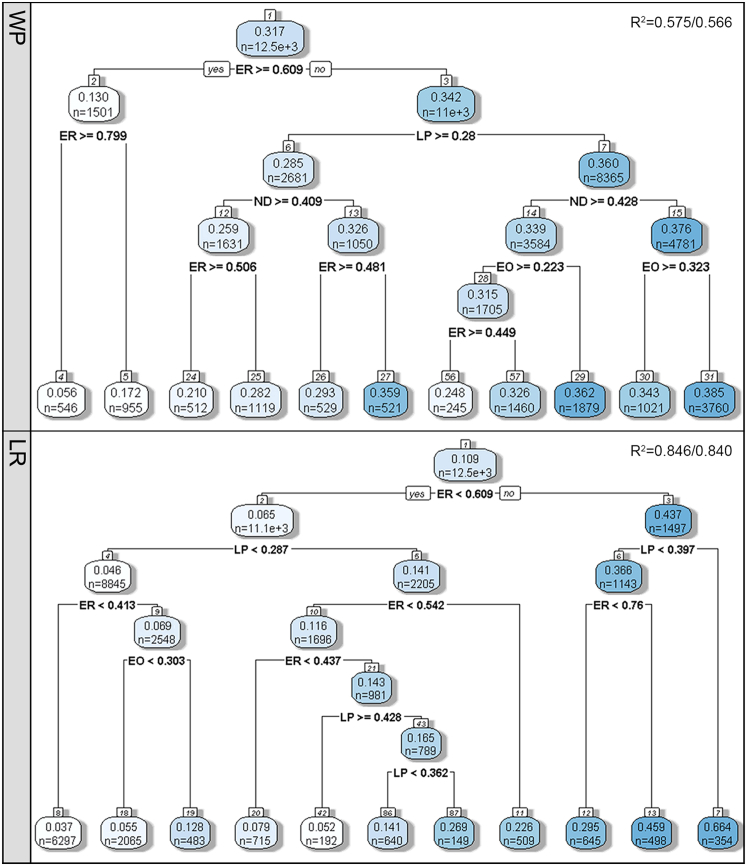
Decision trees for WP and LR with Health-Risk factors in Shenyang

After fitting decision trees between Health-Risk factors and ecosystem service functions, it was found that, except for the low fitting degree of WP, the fitting degrees of other ecosystem service functions were all good, with values greater than 0.8 (Figures 4 and 5).

The Helath-Risk decision tree of CS consisted of four factors (ER, LP, ND, and EO) and 23 nodes, showing three key interactive combinations (ER/ER + LP/ER + EO + ND + LP). Among all factors, EO and ND were significant at only two nodes (nodes 5 and 10) respectively, had a positive link to CS, where the other nodes involved ER or LP. The critical node threshold was ER = 0.627: cells exceeding this value, CS, increased significantly from an average of 0.155–0.575. If LP was controlled below 0.311, CS increased further to 0.723 and continued to strengthen with higher ER. Comparing nodes 1, 2, 4, and 5, it was shown that when ER was below 0.42, it became the only factor significantly affecting CS, resulting in weak capacity and a serious imbalance in ecosystem services. Comparisons among nodes 3, 6, 11, and 20 indicated that in the LP and ER combination, ER’s positive effect was partly suppressed by LP, yet ER remained the most significant factor in determining CS.

The FP Health-Risk decision tree was composed of three factors (LP, ER, and EO) and 25 nodes. The key interactive combinations were LP/LP + ER/LP + EO/LP + EO + ER. Among these, LP was identified as the key determinant with a critical threshold of 0.218. On either side of this threshold, as LP increased, the average value of FP rose from 0.103 to 0.453. Comparisons among nodes 1, 2, 3, and 13 revealed that LP had different effects on FP around the threshold of 0.382. Specifically, LP was positively correlated with FP when LP was below 0.382, but became negatively correlated once LP exceeded this value. A similar pattern was observed for ER at nodes 5, 28, and 59. However, this did not imply that these two factors showed different trends on either side of the threshold. Instead, these changes in correlation were predominantly attributed to the high correlation coefficient of LP, a topic that would be elaborated in the subsequent discussion on the interactions of factors. The interactions among various factors in the FP decision tree were more intricate than those in other service decision trees. Changes in a single factor, especially those with high correlation coefficients, could trigger cascading effects, thereby influencing the stability and sustainability of the entire ecosystem.

The WY decision tree comprised 27 nodes and ND, ER, and LP factors. The key interactive combinations were ND + ER and ND + ER + LP. Under the suppressive effect of ND (ND = 0.41), the mean WY value declined from 0.418 to 0.265 as ND increased. Overall, ND and ER exhibited monotonic negative relationships with WY, whereas LP behaved anomalously at node 12: When ND was well controlled (ND < 0.(41) and ER was well qualified (ER ≥ 0.603), rising LP did not further reduce WY. Comparing nodes 11 with 13, LP significantly attenuated its own suppressive effect on WY when it was greater than 0.422. Further comparison of nodes 5 and 12—where WY values were similar—revealed that ND influenced WY more strongly than ER. Consequently, effective management should prioritize controlling ND and ER, with ND as the primary regulatory target, to achieve sustained optimization of WY services.

The WP decision tree was built from four driver categories (ER, LP, ND, and EO) with 21 nodes. LP and EO contributed a few critical nodes, and the key interactive combinations were ER, ER + LP + ND, and ER + LP + ND + EO. Overall, WP levels were low, but when ER exceeded the threshold of 0.609, WP performance was sharply suppressed. As ER decreased, the average value of WP increased from 0.13 to 0.342. Overall, the changes in functional values on both sides of the LP, ND, and EO factor nodes were relatively weak, but the key factor ER had a strong inhibitory effect on WP, and as ER increased, its inhibitory effect continued to increase (comparisons of nodes 1, 2, 3/12, 24, 25/13, 26, 27/28, 56, 57).

Compared with other decision trees, the decision tree for LR featured a relatively simple set of significant factors and combinations. It was composed of three factors (ER, LP, and EO) and 21 nodes. The key interactive combinations were ER + LP and ER + LP + EO. Notably, EO appeared only at node 9, while the other nodes were primarily involved in ER and LP. The critical factor and threshold were ER and 0.609. When a cell’s ER value was below this threshold, the average value of LR was only 0.065, but it increased to 0.437 when ER exceeded 0.609, and consistently showed a positive correlation. For LP, at node 21 (threshold 0.428), its correlation with LR shifted. As LP increased, it initially suppressed LR, but then became promotional.

### Marginal effects of single and interactive factors

**Figure 6:**
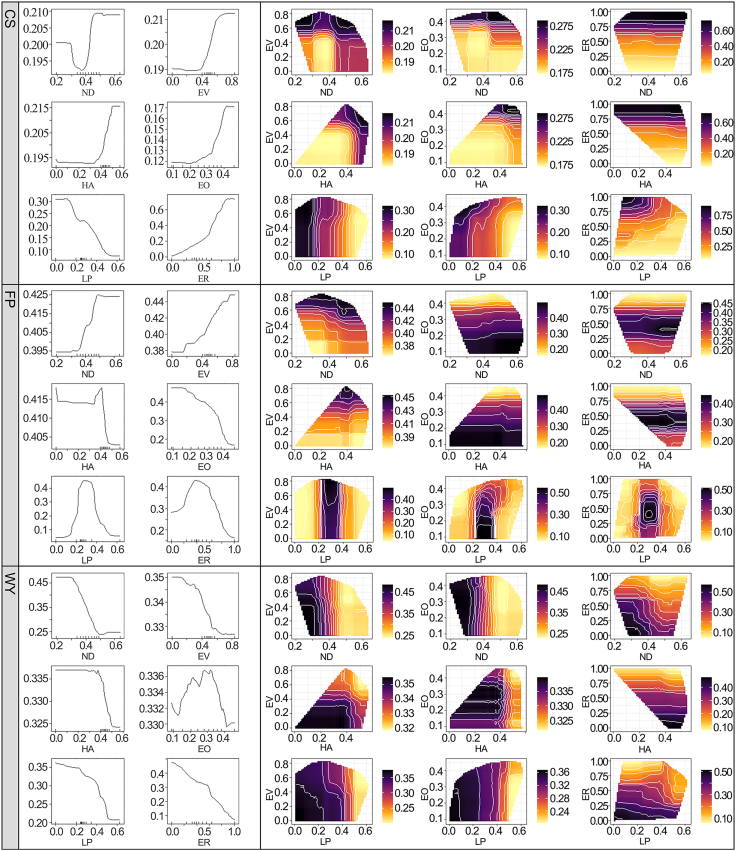
Marginal effects of single and interactive factors for CS, FP, and WY of Health-Risk in Shenyang

**Figure 7:**
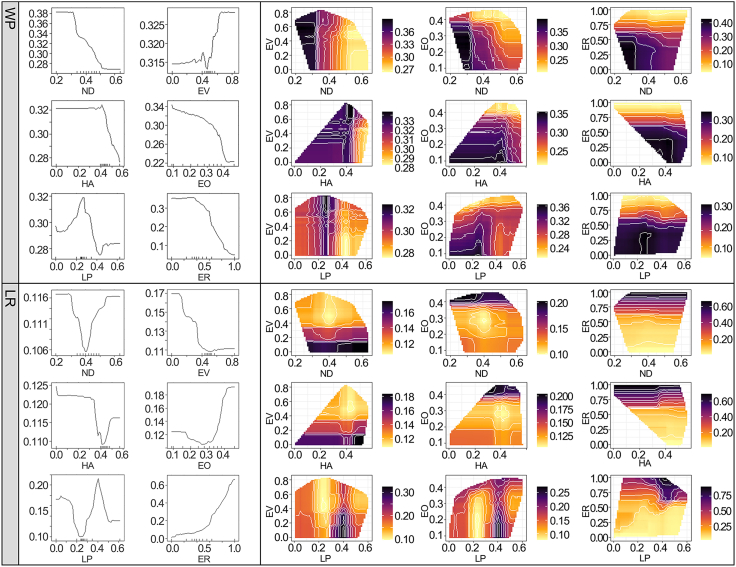
Marginal effects of single and interactive factors for WP and LR of Health-Risk in Shenyang

The study analyzed the value distribution of six Health-Risk factors. It found that HA, LP, and EV had a concentrated value distribution with a high degree of dispersion in D1 (1^st^ decile abbreviated as D1, the same below) and D10, while ND, EO, and ER had a more even distribution, which is consistent with the real-world situation. The marginal effects of Health-Risk factors on ecosystem services, both individually and in combination (Figures 6 and 7), were illustrated in the following figures. The analysis showed that the significant factors affecting the five ecosystem services displayed diverse trends across different threshold ranges.

CS was consistently positively correlated with HA and EV. Before the D5 of ND, it showed a limited negative correlation with CS, but after the 5th percentile, a significant positive correlation emerged. CS showed low sensitivity to these three factors, with marginal effects stabilizing between 0.190 and 0.215. In contrast, LP, EO, and ER had stronger effects on CS, where LP was significantly negatively correlated, with its inhibitory effect decreasing stepwise as it increased. ER and EO were highly positively correlated with CS, with ER showing greater sensitivity than EO, as both showed low initial promotion of CS that strengthened as the variables increased.

Compared with CS, FP exhibited more complex responses to the Health-Risk factors markedly. ND and EV showed low sensitivity and positive correlations with FP, while HA exhibited low sensitivity and negative correlations except before D1 and after D9. The marginal effects of these three factors were stable, ranging from 0.38 to 0.44. LP, EO, and ER had stronger impacts on FP, where EO exhibited a negative association with its inhibitory effect intensifying as EO increased. Both LP and ER followed an initial promotion followed by inhibition: The marginal effect of LP displayed a pronounced bell-shaped distribution, peaking at D8, while significantly suppressing FP before D1 and after D9; ER exerted a significantly stronger inhibitory effect than the promotion it had provided after it reached D3.

The WY function showed weak sensitivity to HA, EV, and EO, with a stable marginal effect of 0.325–0.35, according to the value range for WY of these variables in Figure 6A. The first two were negatively correlated, while the latter showed fluctuating changes. The other three factors were highly sensitive to WY, exhibiting convex or linear relationships, with significant differences in marginal effects. Specifically, LP showed a convex relationship with WY after D2. ND was linearly correlated with WY from D1 to D9, with the service value decreasing from 0.452 to 0.25. After D2, the marginal constraint curve between LP and WY assumed a convex-upward shape. ER exhibited a fluctuating negative correlation, with linear changes before D3 and after D9, and a convex relationship between these deciles.

WP had weak sensitivity to HA, LP, and EV, showing a linear negative correlation with HA, a fluctuating correlation with LP that switched from promotion to inhibition, and a fluctuating positive correlation with the increasing promotion of EV. The marginal effects of these factors were stable between 0.28 and 0.32. In contrast, ND, EO, and ER showed high sensitivity to WP. Across the D1 to D9, all three exhibited a convex negative correlation, accompanied by a significant drop in marginal values.

LR showed high sensitivity only to LP and ER, while the other four factors exhibited low sensitivity with fluctuating correlations and stable marginal differences from 0.01 to 0.06. LP inhibited LR before the D1 and after D9, but showed a nearly linear positive correlation between these deciles, with the peak increasing to twice the valley value. In contrast, ER showed a highly concave positive correlation with LR throughout, with an extreme difference of 0.6.

Univariate analyses had already demonstrated that LP and ER exerted significant influences on ecosystem services via threshold mechanisms, yet these marginal effects were typically confined to specific numerical ranges. Once an interaction framework was incorporated, the above individual effects were rapidly amplified or reversed, transcended the constraints of single thresholds, and converted originally linear marginal responses into nonlinear gains or inhibitions, as detailed in Figure 6B.

In the CS model, interactions were classified as weak (EV + ND, EV + HA), moderate (EO + ND, EO + HA, EO + LP, EV + LP), and strong (all factors combined with ER). Weak interactions yielded stable CS values of 0.19–0.21. Under moderate interactions, CS was strongly suppressed when LP exceeded 0.4, whereas higher EO markedly enhanced the capacity. Strong interactions were dominated by ER: Relationships with ND and HA were largely monotonic, and CS surged once ER surpassed 0.6. Notably, the LP + ER combination produced a pronounced synergy—when LP was below 0.3 and ER above 0.6, CS peaked at 0.75, far exceeding the marginal effects of either factor combination alone. Thus, prioritizing CS in regional planning should exploit the coordinated regulation of ER and LP.

In the FP model, weak interactions were confined to the EV + ND and EV + HA combinations, under which FP values fluctuated narrowly, and functional performance remained stable. Moderate interactions involved every possible pairing among EO, ER, ND, and HA. Within these combinations, as the decrease of EO, FP continued to strengthen. Pairings with ER peaked between 0.25 and 0.60. Strong interactions were restricted to LP combined with EV, EO, or ER. Elevated service values were registered only when LP fell between 0.20 and 0.40, with EO situated between 0.10 and 0.20 and ER between 0.25 and 0.60. No extreme high-function combination emerged under interactive effects in the FP service; nevertheless, the marginal-effect profiles indicated that LP was the principal driver of extreme value. Consequently, targeted interventions should focus on the intervals LP < 0.20 or LP > 0.40.

Weak interactions governing WY were formed by all pairings among EV, EO, and HA, LP. LP exhibited a monotonic negative relationship with these two health factors: WY declined steadily as LP values increased. When HA was paired with EO, maintaining HA below 0.4 was necessary to safeguard WY. In the EV + HA combination, both factors had to be kept below 0.4. Moderate interactions were confined to EV + ND and EO + ND, both of which yielded high values only when ND remained below 0.4. Strong interactions were restricted to ER combined with the three risk factors. Among these, HA had the smallest and most consistently suppressive effect, resulting in lower values than those of ND and LP. The ND + ER and LP + ER interactive combination was more complex: When ND and LP were below 0.4, ER had to be kept below 0.6, whereas if these two factors exceeded 0.4, ER had to be restricted to below 0.25 to maintain high WY. Within these bounds, WY was highest when ER stayed below 0.5. Similar to the FP model, no extremely high-function ensemble emerged under interactive effects. Nevertheless, ER was identified as the principal determinant of low functionality, and targeted management should therefore focus on the interval ER > 0.6.

WP exhibited weak, moderate, and high interactive combinations, which were sequentially composed of EV, EO, and ER in combination with risk factors. The service value ranged for these interactions was 0.27–0.36 for weak, 0.24–0.36 for moderate, and 0.1–0.4 for high interactions. Weak interactions were primarily influenced by the three risk factors. To ensure WP capacity in moderate interactions, the three risk factors needed to be kept below 0.32, 0.45, and 0.3, respectively. In high interactions, ER was the main suppressor, with the ND + ER combination showing a slight interactive promotion effect on WP. When ND was below 0.3 and ER was below 0.45, WP reached its peak value of 0.4, higher than other factor combinations. Additionally, ER could lead to low values of WP. A key measure to address this issue was to control the expansion of units where ER exceeded 0.6. Therefore, in addition to suppressing the high-value areas of ER to ensure the WP function, enhancing the interaction between low-value ND and ER could also promote its improvement, factors that were identified as significant for WY as well.

In the weak interactive relationship, LR were governed by pairwise combinations of EV, EO, and ND, HA; service values remained stable between 0.10 and 0.20, as EV showed a monotonic decline and EO a monotonic rise, combining with risk factors above. EV + LP and EO + LP combinations were moderate interactions. When LP approached 0.40, LR rose to 0.25–0.30, whereas when LP values was in the range of 0.25–0.3 or greater than 0.5, LR was suppressed to 0.10–0.15. High interactions were driven by ER combined with all the risk factors: Under ER + ND and ER + HA interactions, values increased monotonically with ER within 0.20–0.60, whereas ER + LP not only lifted the minimum service value but also pushed the peak to 0.75, contingent on LP > 0.40 and ER > 0.50. LP exerted a marked promotional effect on LR, most pronounced under ER + LP combination: The minimum value improved from 0/0.10 (for single factor) to 0.25, and the maximum value rose from 0.60/0.20 to 0.75. Consequently, areas with high demand for LR should prioritize achieving elevated ER values and implement targeted enhancements in the medium-to-high LP range.

### Mediation effects of health-risk-service

**Figure 8:**
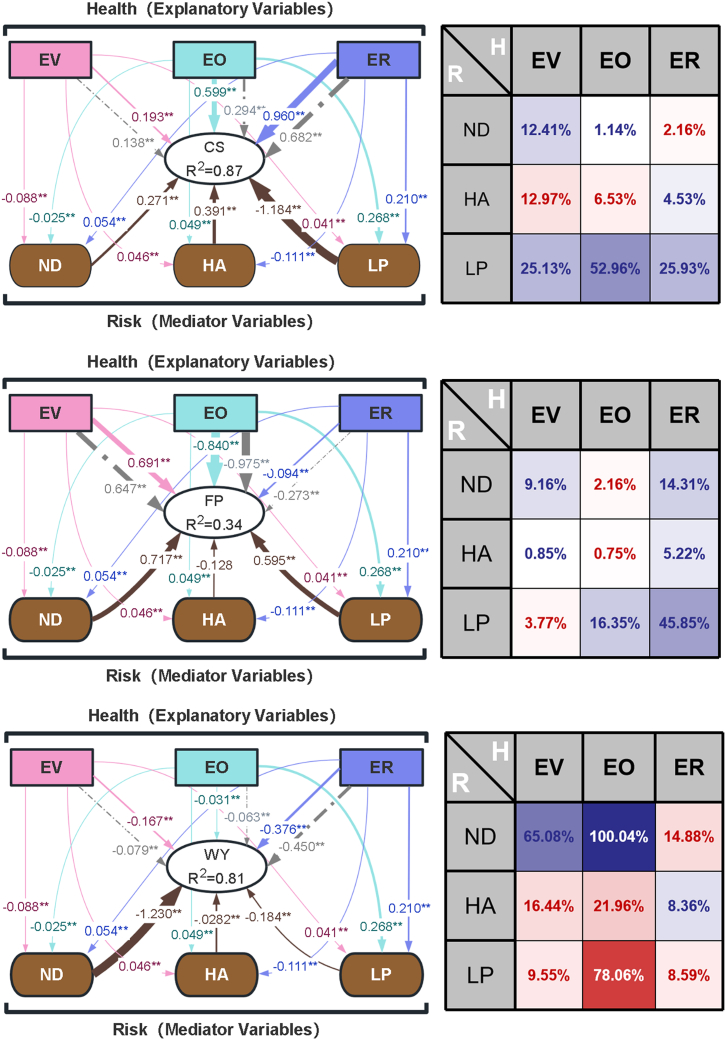
Mediating pathways and effects of Health-Risk factors on CS, FP, and WY in Shenyang

**Figure 9:**
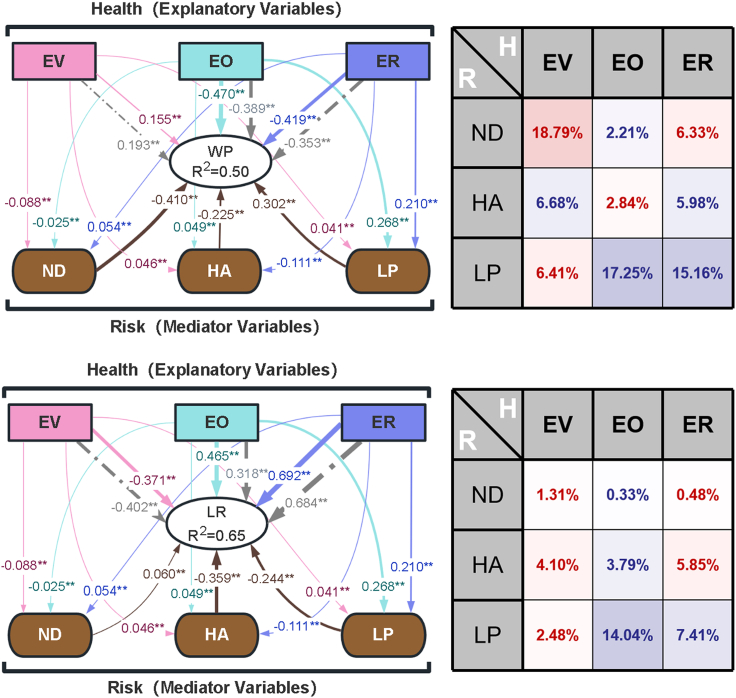
Mediating pathways and effects of Health-Risk factors on WP and LR in Shenyang

In all mediation effect tests, the overall fitting effect of each model was good, with R^2^ values of 0.87 for CS, 0.34 for FP, 0.81 for WY, 0.50 for WP, and 0.65 for LR (Figures 8 and 9) (The solid and dashed lines represent the mediating and total effects, respectively (*p* < 0.01); The number next to the arrow is the standardized pathway coefficient, and the line width is proportional to the strength of the influence; R^2^ represents the variance ratio of the dependent variable explained by the model. Red and blue represent partial mediation effects and masking effects, respectively, while color opacity represents the proportion of effects. Except for the FP mediation chain with HA as the mediator, which failed the significance test, all path coefficients of the interaction between risk and health factors passed the significance test (*p* < 0.01). The impact of health factors on ecosystem services was mainly driven by direct effects, while risk factors, as mediators, exerted differentiated regulatory effects through pathways with different directions. The specific path analysis is as follows.

Regarding total effects, health factors all exerted positive effects on CS, among which ER exhibited the strongest effect (0.682), acting as the core driving factor of CS. When health factors serve as mediator variables, the direct effects of each health factor on CS remained consistent with the total effects, while risk factors exerted differentiated regulatory effects: HA and ND showed positive effects (0.271, 0.391), LP showed an inhibitory effect on CS (−1.184), resulting in masking effects in several pathways. In specific mediation paths, both EV and EO generated significant partial mediating effects through HA, with proportions of 12.97% and 6.53%, respectively, which moderately supplemented the positive effects on CS. Meanwhile, EV and EO produced masking effects through ND and LP, among which the masking proportions through LP were relatively high (25.13%, 52.96%), significantly weakening the promotion effects on CS. The effects of ER on CS were largely mediated by LP (25.93%) and to a lesser extent by HA (4.53%). Although ND presented a partial mediating effect, its proportion was extremely low (2.16%), indicating a limited contribution.

For FP, EV exerted a positive effect (0.647), while EO and ER showed negative effects (−0.840, −0.094), with ER having the extremely low inhibition. When health factors served as mediator variables, the direct effects of health factors on FP were enhanced, and risk factors showed differentiated effects: HA inhibited FP (−0.128), while ND and LP promoted it (0.717, 0.595), forming a mutual antagonistic regulatory pattern. EV formed weak mediating effects on FP, generating a positive partial mediating effect through LP (3.77%), and masking effects through ND and HA (9.16%, 0.85%). EO mainly exhibited a masking effect through LP (16.35%), which substantially weakened the inhibitory effect. Meanwhile, weak mediation effects were observed for ND and HA (2.16% and 0.75%, respectively). ER showed masking effects through all risk factors (14.31%, 5.22%, and 45.85%, respectively), imposing a significant reverse regulation on the direct inhibitory effect.

For WY, all health factors exhibited negative total effects on WY, among which ER showed the most prominent inhibitory effect (−0.450). When risk factors served as mediator variables, all risk factors exerted negative effects to varying degrees (−1.230, −0.282, −0.184). In specific paths, both EV and EO generated strong masking effects through ND, accounting for 65.08% and 100.04%, weakening or even offsetting their negative effects on WY. Meanwhile, EV formed partial mediating effects through HA and LP, with proportions of 16.44% and 9.55%, further reinforcing the inhibitory effect. The partial mediating effects of EO through HA and LP were significantly stronger than those of EV, accounting for 21.96% and 78.06%, which intensified the inhibition on WY. Both the mediating and masking effect ratios of ER with risk factors were relatively low, showing partial mediating effects through ND and LP (14.89%, 8.59%) and a masking effect through HA (8.36%).

Regarding the total effects, EV showed a positive effect on WP (0.193), while EO and ER exhibited negative effects (−0.389, −0.353), with the inhibitory intensity significantly higher than the promoting effect. The direct effects of health factors on WP remained consistent with the total effects, but the mediating effects of risk factors on WP diverged noticeably: LP showed a positive effect of 0.302, whereas HA and ND showed negative effects (−0.225, −0.410), resulting in distinct mediating characteristics through different paths. In specific pathways, EV formed partial mediating effects through LP and ND, accounting for 6.41% and 18.79% respectively, strengthening the positive relationship with WP; meanwhile, EV generated a masking effect through HA (6.68%), weakening the effect of EV on WP. EO exhibited a limited partial mediating reinforcement through HA (2.84%); in contrast, masking effects through LP and ND accounted for 17.25% and 2.21% substantially weakening the inhibitory effect on WP. The partial mediating effect of ER through ND only supplements the relationship for 6.33%; meanwhile, masking effects through HA and LP accounted for 5.98% and 15.16% respectively, weakening the inhibitory effect of ER on WP.

For LR, the effect degree of the three health factors was relatively balanced, with total effect values of −0.402, 0.318, and 0.684, respectively. The directions of risk factors differed significantly: ND showed a weak positive effect (0.060), while HA and LP exhibited significant inhibitory effects (−0.359, −0.244), resulting in substantial differences in mediating effects through pathways. In specific paths, EV formed partial mediating effects through all risk factors with low proportions (2.48%, 1.31%, and 4.10%), providing a slight supplement to the inhibitory effects on LR. EO showed masking effects through all risk factors, but only the masking effect of LP substantially offset the positive effect (14.04%). LR had partial mediating effects through HA and ND, accounting for 5.85% and 0.48%, strengthening its positive relationship with LR; meanwhile, a relatively strong masking effect was served through LP (7.41%).

### Key thresholds for spatial control in typical areas

**Figure 10:**
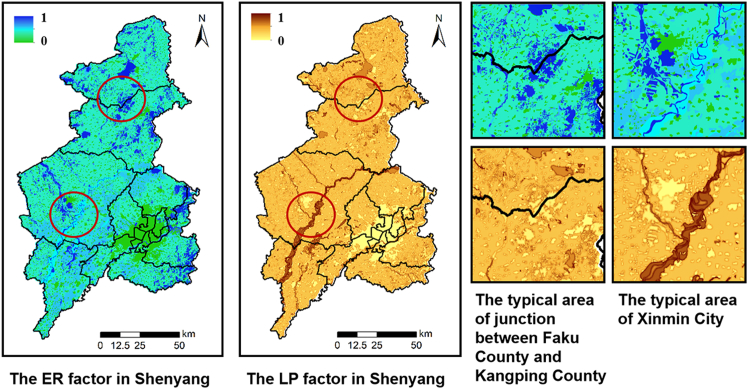
The ER and LP of Typical areas for spatial control in Shenyang

As a key ecological buffer between Shenyang’s urban and rural areas, the suburban area (Figure 10) faces dual pressures of urban sprawl and agricultural intensification. Long-term monitoring shows its current status: LP = 0.28 (slightly exceeding the threshold of 0.(218) and ER = 0.52 (approaching the critical value of 0.6). This “quasi-critical” state determines that neither single LP regulation nor independent ER enhancement can achieve sustainable management; instead, a “threshold-based synergy strategy” integrating “strict boundary control + hierarchical restoration” is required.

The primary contradiction in this zone lies in the “fragmented occupation” of ecological space: scattered construction land (accounting for 18% of the zone) and high-intensity farmland (62%) have split the original continuous grassland and woodland into patches smaller than 5 hm^2^, leading to a 28% reduction in surface runoff regulation capacity. Meanwhile, the LP index is pushed above the threshold by “low-efficiency expansion” (e.g., abandoned rural homesteads and small-scale industrial parks), while ER remains stagnant due to the lack of connected ecological corridors. Based on the “LP-ER synergy window” (LP ≤ 0.218, ER ≥ 0.(6) defined in this study, the intervention is divided into two phases with clear priorities, combining policy instruments and technical measures.

1. Phase 1 (1–3 years): LP threshold control with “spatial reconstruction.”

Precise LP reduction. In accordance with “Overall Plan for Land and Space of Shenyang City (2021–2035).” For 2.3 × 10^3^ hm^2^ of low-efficiency construction land identified via remote sensing, 60% will be converted to ecological land (forests and grassland) and 40% to concentrated industrial parks (to avoid scattered expansion). This is expected to reduce LP from 0.28 to 0.22, returning it to the threshold range.

Ecological boundary locking with “dual red lines” (Two mandatory protection boundaries in China: a. Ecological Protection Red Line: Safeguards critical ecosystem functions and ES; b. Permanent Basic Farmland Red Line: Ensures food security by protecting high-quality arable land.). Demarcate the “construction-ecology boundary” using high-precision GIS spatial analysis, and install electronic monitoring stakes in key sections around the Shenyang National Forest Park, Liaoning, and Hunhe National Wetland Park to prohibit construction land from encroaching on ecological corridors wider than 100 m. This measure references the successful experience of state forestry farms' protection red line management, which has reduced illegal land occupation by 75% in similar zones.^33^

2. Phase 2 (4–6 years): ER enhancement with “corridor-network construction”

Key corridor restoration. Construct three east-west ecological corridors (width 50–150 m) along the terrain, connecting the Hunhe Wetland, Qipanshan Scenic Area, and Liaozhong Grassland. The restoration uses native species to avoid biological invasion, with a target of increasing vegetation coverage from 42% to 65%. According to regional restoration models,^34^ this will enhance ER by 0.12–0.15, reaching 0.64–0.67.

Agro-ecological synergy. Promote “ecological farming” in 80% of the farmland within the zone, such as setting up 10-meter-wide grass strips between crop fields and reducing chemical fertilizer use by 30%. This measure not only improves soil CS capacity (increasing ER by an additional 0.03) but also increases farmers’ income through organic crop certification—addressing the conflict between ecological protection and economic development in the transition zone.

The effectiveness of the above scenario will be monitored through the “LP-ER dynamic early warning system” established in this study: Real-time updates of LP and ER values will be conducted using annual satellite remote sensing data and quarterly field surveys. To ensure implementation, the Shenyang Municipal Government will incorporate the scenario targets into the “Ecological Civilization Construction Assessment System” of district governments, with a 30% weight in the performance evaluation. Financial support will rely on the *“ecological compensation fund for urban-rural transition zones*” (500 million yuan/year), with 70% used for ecological restoration and 30% for farmers’ compensation.

## Discussion

### Integration and exploration of the health-risk-service triad framework

Traditional research on ecosystem security mainly focused on pairwise relationships between ecological risk, ecosystem health, and ecosystem services, with some studies introducing other influencing factors.^35^^,^^36^^,^^37^ Building on this, this study integrates these three dimensions into a unified Health-Risk-Service triad framework, clarifying their causal relationships, verifying nonlinear regulatory effects of Health-Risk factor thresholds on services, and delineating their interaction and mediation pathways. Aligned with the SES framework,^38^ this study refines the ecological dimension by identifying quantifiable thresholds,^39^ extending prior understanding and overcoming single-index limitations.^40^ Mediation analysis further shows that ecosystem health affects services primarily through direct effects, while ecological risk acts through partial mediating and masking effects with service-specific heterogeneity, confirming risk factors regulate services in specific ways.

By integrating fragmented pairwise relationships into a verifiable triadic model, this study provides operational regulatory pathways for different regions and services, offers theoretical guidance for core factor interactions and service synergies, and lays a foundation for multi-scale scenario simulations and adaptive management, transforming synergistic service enhancement from an academic concept into a practical tool for sustainable development.

### Synergistic regulation: Application of interaction and mediation effects

Based on the research results of interaction effects and mediation mechanisms, a closed-loop application system of “health foundation building-risk transmission-service optimization” is constructed, with health indicators as the core driver and risk factors as the transmission carrier of ecosystem services, breaking the limitation of traditional single-factor regulation.

First, prioritize improving ecosystem health, focus on the direct driving role of health factors, and leverage the interaction between health and risk to ensure that health improvement effectively translates into service enhancement; Second, precisely target mediation paths and implement differentiated regulation based on the mediation pathways of different services, balancing the interaction and antagonistic/synergistic effects between Health-Risk factors to maintain service stability; Finally, combine the synergistic impact of health and risk and the differentiated service responses to implement service-oriented classified regulation, aiming to reduce service loss and achieve coordinated improvement of multiple ecosystem services.

This perspective is in consensus with recent research conclusions by some scholars. For instance, Valsin et al. conducted that reducing forest fragmentation (lowering LP) and increasing forest cover (raising ER) can significantly enhance ecosystem services related to forest and water quality through a cost-effectiveness analysis of payments for tree planting and forest management.^41^ Additionally, in Quyang County of the Taihang Mountains in Hebei Province, China, ecosystem service evaluation guided by landscape ecology theory improved land-use planning while enhancing the comprehensive value of ecosystem services according to *Hebei Academy of Social Sciences 2022*.^42^ This aligns highly with the “ecological security pattern-ecological restoration” synergistic model.^43^^,^^44^

### Generalizability: Framework universality and differences

The Health-Risk-Service machine learning framework developed in this study exhibits inherent universality, stemming from its alignment with the core tenets of the SES framework and the “function-structure-service” cascade of ecosystems—a fundamental theoretical basis for regional ES assessment. By integrating three core dimensions of regional ES (ecological risk, ecosystem health, and ecosystem services) and employing interpretable machine learning models (e.g., decision trees and random forests), this framework identifies regulatory relationships among key variables without over-reliance on region-specific parameters, thereby enhancing its cross-regional applicability. Consequently, it can be adaptively applied to arid or tropical urban areas with minimal modifications:

For arid regions, ecological risk indicators can be supplemented with the temperature vegetation dryness index, and ecosystem health metrics can prioritize lake surface areas—consistent with the water-limited nature of arid ecosystems^45^; for tropical regions, indicators such as surface and air temperature can be incorporated to optimize the indicator system, while the overall analytical logic and model structure remain unaltered.^46^

In contrast, numerical thresholds and mediating effects represented by ER exhibit significant context dependence, which is mainly constrained by different types of factors. First, climate zones directly determine the baseline of ecological resilience (ER): With scarce water resources, the ER threshold for maintaining basic ecosystem services may be lower and mediating effect of ND may be more effective in arid regions (e.g., In karst landform areas, the key thresholds of ER for WY and WP are 0.4 and 0.5 respectively, which are significantly lower than the results of this study),^47^ which is highly consistent with the characteristics of arid ecosystems. Second, the stage of economic development modulates the threshold through human disturbance intensity: In industrialized cities with high-intensity land development (similar to Shenyang), the ER threshold of 0.6 reflects the balance between industrial land occupation and ecological conservation.

Notably, the overall analytical logic and model structure remain unaltered across these adaptations, underscoring the framework’s transferability. This universality is further supported by empirical evidence from arid and tropical ecosystem studies, where integrating core ecological dimensions (risk, health, services) has proven effective for identifying context-independent regulatory mechanisms.^48^

### Limitations of the study

It should be objectively stated that while this study advances innovations in dynamic ES assessment methodologies, it still retains room for refinement due to constraints related to data accessibility and the phased scope of the research design. In terms of the temporal dimension, the current research primarily relies on cross-sectional data from a single time point (2020) for analysis. While this approach effectively delineates the correlational characteristics and mediating effects between ecosystem services and Health-Risk factors during the study period, it fails to incorporate long-term time-series data to track the cumulative impacts of land use change, climate variability, and other processes on the Health-Risk-Service system. Regarding the selection of ecosystem service types, the quantification of cultural services predominantly depends on landscape metrics and POI data, lacking support from residents’ subjective perception surveys. This limitation may introduce certain biases into the assessment results of such services. Additionally, in terms of driving factor coverage, the study focuses on core ecological risk and health factors to construct the analytical framework, with insufficient consideration of certain socio-economic drivers—including policy interventions (e.g., adjustments to regional ecological protection red lines and special policies for cultivated land protection) and industrial structure transformation. In terms of assessment scale and model application, the study uniformly adopts a 1 km grid as the basic assessment unit and does not conduct further multi-scale comparative validation. This hinders the clarification of the scale dependence of threshold effects and cross-scale transmission characteristics. These limitations warrant further optimization and improvement in subsequent research.

Based on the existing foundation and identified areas for the improvement of this study, subsequent research can be further deepened and expanded across multiple dimensions to more comprehensively and accurately support regional ES management practices. In terms of temporal dynamic research, multi-temporal data on land use, climate, and socio-economics from 2000 to 2025 can be integrated to construct a long-panel analytical framework. The focus will be on tracking the evolutionary trajectory of the Health-Risk-Service system and revealing the dynamic variation patterns of key thresholds over time. Furthermore, building on the LP-ER synergy window identified in this study, scenario simulation research (e.g., under different land use planning and ecological restoration project scenarios) can be conducted to predict the evolutionary trends of ecosystem services across various contexts. This will further quantify the long-term cumulative responses and lagged feedback mechanisms of ecosystem services, providing a temporal dimension of scientific basis for formulating dynamically adaptive ecological management strategies. By constructing a “natural-social” dual-dimensional driving model, the combined impacts of the synergistic/antagonistic effects of human regulation and natural factors on ecosystem service thresholds can be analyzed. For instance, additional socio-economic drivers can be incorporated, such as policy intervention variables, industrial structure transformation, and population mobility characteristics. In optimizing the assessment scale and advancing model performance, multi-scale nested assessments can be implemented to analyze the scale dependence of key thresholds and interactive effects, while clarifying cross-scale transmission paths and regulatory nodes. Concurrently, deep learning models can be introduced to enhance the capacity to capture the long-term lag effects of factor interactions, thereby further overcoming the limitations of traditional machine learning methods.

## Resource availability

### Lead contact

Requests for further information and resources should be directed to and will be fulfilled by the lead contact, Xiaofei Xu PhD (xu_xiaofei_neu@163.com).

### Materials availability

This study did not generate new unique reagents.

### Data and code availability

• **Data:** Data reported in this paper will be shared by the lead contact upon request.
• **Code:** This paper does not report original code.
• **Other items:** Any additional information required to reanalyze the data reported in this paper is available from the lead contact upon request.

## Acknowledgments

This study was supported by the 10.13039/501100001809National Natural Science Foundation of China (41871162) and the 10.13039/501100005047Natural Science Foundation of Liaoning Province (2025-MS-029).

## Author contributions

Conceptualization, software, methodology, and writing – original draft, X.X.; supervision and writing – review, editing, and funding acquisition, C.X.; investigation, data curation, supervision, and writing – review and editing, Y.G. All authors have read and agreed to the published version of the manuscript.

## Declaration of interests

The authors declare no competing interests.

## STAR★Methods

### Key resources table

**Table:** 

REAGENT or RESOURCE	SOURCE	IDENTIFIER
**Deposited data**		

Land use data	Resource and Environmental Science Data Platform	https://www.resdc.cn/
Digital elevation model	National Aeronautics and Space Administration	https://earthdata.nasa.gov/
Precipitation data	National Earth System Science Data Center	https://www.geodata.cn/
Evapotranspiration data	National Tibetan Plateau Science Data Center	https://data.tpdc.ac.cn/
NDVI	NASA MYD13A1 Version 6 product	https://nesdc.org.cn/
Plant available water fraction	SoilGrids from World Soil Information	https://www.fao.org/
Watershed	National Cryosphere Desert Data Center	https://code.earthengine.google.com/
Population	National Tibetan Plateau Data Center	https://data.tpdc.ac.cn/
Points of interest (POI)	OpenStreetMap	http://download.slipo.eu/results
Administrative boundaries and road network data	OpenStreetMap	http://download.slipo.eu/results
Socio-economic data	Statistical Yearbook	https://www.shenyang.gov.cn/zwgk/fdzdgknr/tjxx/tjnj/

**Software and algorithms**		

ArcGIS 10.7	ESRI	https://www.arcgis.com/index.html
Invest 3.11.0	Natural Capital Project	https://naturalcapitalproject.stanford.edu/software/invest
MaxEnt 3.4.1	Maxent Software for modeling species niches and distributions	https://biodiversityinformatics.amnh.org/open_source/maxent/
Fragstats 4.3	University of Massachusetts	https://www.umass.edu
R studio 4.4.1	The R Project for Statistical Computing	https://www.r-project.org
Stata 18.0	Announcing Stata 18	https://www.stata.com/

### Experimental model and study participant details

This study does not involve any experimental animals, human participants, plants, microbe strains, cell lines, primary cell cultures used in the study.

### Method details

#### Study area

Shenyang City, a key city in Northeast China, encompasses 10 districts, 2 counties, and 1 county-level city. As of 2020, Shenyang had a total land area of 12,860 km^2^, with an urban area spanning 5,116 km^2^. The urban population was 6.741 million, reflecting an urbanization rate of 81.0%.

Between 2000 and 2020, Shenyang’s urban population growth rate reached 25.9%, urban construction land area increased from 140.9 km^2^ to 197.6 km^2^, and GDP rose from 1.1 × 103 billion yuan to 6.6 × 103 billion yuan. However, rapid population growth, land urbanization, and economic development have inevitably consumed vast natural resources in Shenyang, leading to frequent issues such as biodiversity loss, intensified heat island effects, water scarcity, soil erosion, and declining living environment quality. These problems have severely disrupted the regional ecosystem and ecological security patterns.

#### Data sources

Land use data were acquired from the Resource and Environmental Science Data Platform, and the geospatial data cloud provided the digital elevation model along with additional information (see key resources table). Using the first-level classification system, we reclassified the raw data into six categories: cultivated land, forest, grassland, waters, construction land, and unused land, and the classified and processed data were mosaicked and cropped by applying vector boundaries, with a spatial resolution of 1 km × 1 km.

Given that the 1 km grid scale is the smallest unit for ecological management in China, this study conducted ecosystem assessments at this scale.

#### Research methods

##### Selection of health-risk-service factors

Based on China’s ecological protection policies such as the *Guidelines for Resource and Environmental Carrying Capacity and Territorial Space Development Suitability Evaluation (2020 edition)* and typical ecological issues in Shenyang, this study selected five ecosystem services for evaluation, covering four major types: supporting, regulating, provisioning, and cultural services. The specific assessment methods were listed below (see table below).

**Table:** Method of calculating ecosystem services

Ecosystem Services	Indicator Definitions and Calculation Formula
CS	Green vegetation absorbs CO_2_ and water through photosynthesis, sequestering carbon within plants.^49^ Calculated using the InVEST Carbon Storage and Sequestration module. (Equation 1) $$\begin{array}{c} {CS}_{i}=C_{above}+C_{below}+C_{soil}+C_{dead} \end{array}$$ $C_{above}$ represents above-ground biogenic carbon stock, $C_{below}$ represents below-ground biogenic carbon stock, $C_{soil}$ represents soil carbon stock, and $C_{dead}$ represents the dead organic carbon stock.
FP	Provision of staple crops like rice by ecosystems. As NDVI was strongly correlated with grain yield, the study calculated NDVI downscale grain yield statistics, distributing Shenyang’s food prodction to individual cropland pixels.^50^ (Equation 2) $$\begin{array}{c} {\text{FP}}_{i}=\frac{{\text{NDVI}}_{i}}{{\text{NDVI}}_{mean}}\times f \end{array}$$ ${\text{NDVI}}_{i}$ represents the NDVI value of grid i on cultivated land in Shenyang City, ${\text{NDVI}}_{mean}$ refers to the average NDVI value of cultivated land in Shenyang City, and f represents the annual grain yield of Shenyang City (kg/hm^2^). In this formula f=7576, which is consistent with the statistical data from Shenyang’s 2020 Planning Yearbook.
WY	Capacity of water ecosystems to provide freshwater resources to nature and human societies.^51^ Calculated using the InVEST Water Yield module. (Equation 3) $$\begin{array}{c} {\text{WY}}_{i}=\left(1-\frac{{\text{AET}}_{i}}{P_{i}}\right)\times P_{i} \end{array}$$ ${\text{AET}}_{i}$ represents the actual evapotranspiration per year of raster grid i, $P_{i}$ symbolizes the typical yearly precipitation of raster grid i.
WP	Simulates spatial migration of nutrients like nitrogen and phosphorus, estimating retention efficiency.^52^ Calculated using the InVEST Nutrient Delivery Ratio module. (Equation 4) $$\begin{array}{c} {WP}_{d}=X_{\exp}=\sum_{i}Modified{load}_{\left(x,i\right)}\times {NDR}_{i} \end{array}$$ $X_{\exp}$ represents nitrogen exported to water bodies; $Modified{load}_{\left(x,i\right)}$ represents nitrogen loading from grid I adjusted for local conditions; ${NDR}_{i}$ is Nitrogen output rate.
LR	Potential of terrestrial and aquatic landscapes to provide recreational services. Calculated using the MaxEnt model, with scenic spots as samples and environmental variables like elevation, slope, and land-use type.^53^ (Equation 5) $$\begin{array}{c} {\text{LR}}_{i}=\frac{S_{park_{i}}+S_{square_{i}}+S_{scenic_{i}}+S_{botan_{i}}+S_{zoo_{i}}+S_{memo_{i}}+S_{{herit}_{i}}+S_{{aqua}_{i}}}{8} \end{array}$$ $S_{park_{i}},S_{square_{i}},S_{scenic_{i}},S_{botan_{i}},S_{zoo_{i}},S_{memo_{i}},S_{{herit}_{i}},S_{{aqua}_{i}}$ respectively represent the distribution indices of parks, squares, scenic spots, botanical gardens, zoos, memorial parks, world heritage sites, and aquariums in grid i.

In this study, the accuracy of the ecosystem service assessment was verified by comparing the model simulation results with existing literature data, and the verification results are as follows(see table below).

**Table:** Simulation Results and Literature Data

Ecosystem Service	Simulation Data	Literature Data
CS	Average: 4.6×10^6^ t	According to the 30m-Resolution Remote Sensing Monitoring and Simulated Spatial Dataset of Carbon Storage in The Grain for Green Project Areas of Northeast China in 2020,^54^ the Carbon Sequestration of the 2020 years is 5.0×10^6^ t, which shows a low error margin relative to the simulation data.
FP	Average: 7.6 × 10^3^ kg/ha Annual total: 4.60 × 10^6^ t	According to the *2021 Statistical Yearbook of Shenyang City*, the Food Production output of the 2020 years is 4,094,285 tons, which shows a low error margin relative to the simulation data.
WY	Annual total:2.0 × 10^10^ m^3^	According to *the 14th Five-Year Plan for Reclaimed Water Utilization of Shenyang City issued in 2021*, the multi-year average total water resources of Shenyang City stands at 2.356 billion cubic meters, which shows a low error margin relative to the simulation data.
WP	Average: 3.8 × 10^4^ t	To address the regional inapplicability of the InVEST model’s default parameters, we referred to published similar studies in the study area and surrounding regions to perform local calibration of core parameters (e.g., biophysical Tables, Threshold Flow Accumulation and Borselli K Parameter).
LR	Average: 0.117	The average index was not obtained, yet the spatial distribution characteristics of the simulation data in this study are consistent with those of other relevant studies.^55^

Ecosystem health is reflected through EV, EO, and ER.^56^ EV, measured by NPP (Net primary productivity) represents the metabolic capacity of ecosystems^25^^,^^57^; EO reflects ecosystem structural complexity, derived from landscape heterogeneity, connectivity, and habitat connectivity^58^; ER, closely tied to biodiversity, is represented by habitat quality indices.^59^ The calculation process is detailed in the table below.

**Table:** Components and Quantification of Ecosystem Health

Element Layer	Indicator Layer	Calculation Process
EV	Net primary productivity	Utilizing the MOD17A3 global NPP data released by the National Aeronautics and Space Administration (NASA), this data employs the BIOME-BGC model and light use efficiency model to estimate regional annual NPP and performs normalization processing.
EO	Landscape heterogeneity	Using the moving window method in Fragstats software, calculated landscape diversity (SHDI) and landscape fractal dimension (AWMPFD), and summed up with weights of 0.25 and 0.1 respectively.
	Landscape connectivity	Using the moving window method in Fragstats software, calculated landscape contagion index (CONTAG), and summed up with weights of 0.35.
	Habitat connectivity	Using the moving window method in Fragstats software, calculated patch fragmentation (PD) and patch cohesion (COHESION) of forest land, grassland, and water, and summed up with weights of 0.07 and 0.03 respectively.
ER	Habitat quality	Calculating the Habitat Quality module of the InVEST model. The half-saturation constant k is 1/2 of the maximum value of habitat degradation, which is taken as 0.097.

Ecological risk refers to the multiple pressures exerted by ND, HA, and LP on ecosystems.^60^^,^^61^^,^^62^ Considering frequent droughts and soil erosion in the study area, this study measured ND using drought and geological risk indicators^63^; HA was reflected through human footprint, urban expansion, and heat island effects^64^; LP was characterized by the product of landscape disturbance and landscape vulnerability indices.^65^ The calculation process is detailed in the table below.

**Table:** Components and Quantification of Ecological Risk

Element Layer	Indicator Layer	Calculation Process
ND	Drought risk	Equally weighted average of the percentage of precipitation anomaly in December (representing meteorological drought), soil moisture index (representing soil drought), and normalized difference water index (representing vegetation drought) to construct a comprehensive index.
	Geological risk	Calculate the slope index based on the digital elevation model, set values below 2° as risk-free, values above 25° as maximum risk, and perform normalization for values between 2° and 25°
HA	Human footprint	Equally weighted average of normalizing the three indicators of population density, land use type, road accessibility,and assigning values to land use types, to construct a comprehensive index.
	Urban expansion	Based on the Landsat-8 surface reflectance imagery provided by the Google Earth Engine platform, calculations are performed according to the normalized dryness index formula.
	Heat island effect	Based on the Landsat-8 surface reflectance imagery provided by the Google Earth Engine platform, the surface temperature index is inverted
LP	Landscape disturbance	Using the moving window method in Fragstats software, calculated landscape fragmentation (PD), landscape isolation (SPLIT), and landscape fractal dimension (DIVISION), and summed up with weights of 0.5, 0.3, and 0.2 respectively.
	Landscape vulnerability	Based on existing research findings, ranked and scored land use types, and then subjected to summation and normalization processing. The final ranking is as follows: unused land (0.25) > waters(0.18) > cultivated land (0.14) > grassland (0.11) > forest (0.07) > construction land (0.04)

#### Key threshold identification based on decision trees

Common methods for analyzing driving factors of ecosystem services include traditional regression, geographically weighted regression, and machine learning algorithms.^66^^,^^67^ However, influencing factors often affect ecosystem services nonlinearly, with varying correlation patterns across different service types in reality.^68^ Traditional linear regression methods may thus yield biased results.^69^ In contrast, decision trees (DT) classify data based on different principles, accurately identifying nonlinear relationships, key thresholds, and turning points where effect strength or direction changes significantly.^70^

This study used the R package *rpart* to perform classical decision tree-based analysis of driving factors, with Health-Risk factors as explanatory variables and ecosystem services as response variables. The dataset was randomly split into 80% training sets and 20% testing sets. Pre-pruning and post-pruning were performed on the data, with the minimum number of samples required for node splitting and the minimum number of samples for leaf nodes set to 500 and 100, respectively, and the complexity parameter (cp) constrained to 0.005. Classification hypotheses with P-values >0.05 were rejected, and the model iterated until all splits were insignificant or the minimum node size was reached.

#### Marginal effects analysis based on random forests

Random Forests (RF), an ensemble learning-based machine learning algorithm, combines multiple decision trees to improve prediction accuracy. They are widely used for classification, regression, and uncovering relationships between explanatory and response variables.^71^

This study used the R package *randomForest* to analysis the interaction of driving factors, with Health-Risk factors as explanatory variables and ecosystem services as response variables. The data were split into 80% training sets and 20% testing sets as above to construct RF classification models. The number of decision trees was set to 100, 3 features were randomly selected for splitting at each node of every tree, and the proximity matrix between samples was calculated. Partial dependence plots (PDP) were used to reveal nonlinear relationships and analyze marginal effects under single and combined driving factors.

#### Estimation of Mediation effects

In summary, ecological risk, ecosystem health, and ecosystem services interact with each other to jointly form a causal chain underlying regional ecological security. Based on the research hypotheses proposed above, five ecosystem services were separately selected as Explanatory Variables, ecosystem health indicators as explained variable, and ecological risk as the mediating variable; mediation effect analysis was performed using Stata 18.0, and the bootstrap method with 1000 replications was adopted to estimate and test the model path coefficients and coefficient of determination (R^2^).

### Quantification and statistical analysis

All statistical analyses were performed in ArcGIS 10.7, Invest 3.11.0, MaxEnt 3.4.1, Fragstats 4.3, R studio 4.4.1 and Stata 18.0. ArcGIS was responsible for basic spatial data processing, administrative boundary extraction, land use interpretation and spatial pattern mapping, supporting the overall spatial analysis framework of this study. The InVEST model was applied to evaluate ecosystem service functions and quantify the spatial differentiation characteristics of ecological service supply and demand. MaxEnt was utilized to simulate LR service with scenic spots as samples and environmental variables like elevation, slope, and land-use type. Fragstats 4.3 was adopted to calculate a series of landscape pattern indices and further reveal the landscape structural characteristics behind land use transitions. R Studio 4.4.1 served for data collation, spatial statistical calculation and visual plotting of analytical results, while Stata 18.0 was used to conduct quantitative statistical analysis, correlation testing and mechanism regression modeling. All raw data were strictly screened and quality-controlled to ensure the reliability and rationality of subsequent model simulation and statistical analysis results.
